# Composite Sorbents Based on Polymeric Se-Derivative of Amidoximes and SiO_2_ for the Uranium Removal from Liquid Mineralized Media

**DOI:** 10.3390/gels11010014

**Published:** 2024-12-27

**Authors:** Anna I. Matskevich, Konstantin V. Maslov, Veronika A. Prokudina, Daria D. Churakova, Vladimir V. Korochencev, Oleg Yu. Slabko, Evgenij A. Eliseenko, Eduard A. Tokar’

**Affiliations:** 1 Institute of Natural Sciences and Technosphere Safety, Sakhalin State University, 693000 Yuzhno-Sakhalinsk, Russia; mysmatskevich@mail.ru (A.I.M.); maslov.kv@dvfu.ru (K.V.M.); veronikaprokudina2002@mail.ru (V.A.P.); elka2508@bk.ru (E.A.E.); 2 Institute of High Technologies and Advanced Materials, Far Eastern Federal University, 690922 Vladivostok, Russia; dawa.cyratava@mail.ru (D.D.C.); slabko.oyu@dvfu.ru (O.Y.S.); 3 Institute of Chemistry, Far Eastern Branch, Russian Academy of Sciences, 690022 Vladivostok, Russia; korochentsev.vv@dvfu.ru

**Keywords:** sorption, amidoxime, uranium, 2,5-oxadiazoles, polymers

## Abstract

A new composite material with enhanced sorption-selective properties for uranium recovery from liquid media has been obtained. Sorbents were synthesized through a polycondensation reaction of a mixture of 4-amino-N’-hydroxy-1,2,5-oxadiazole-3-carboximidamide (hereinafter referred to as amidoxime) and SiO_2_ in an environment of organic solvents (acetic acid, dioxane) and highly porous SiO_2_. To establish optimal conditions for forming the polymer sorption-active part and the synthesis as a whole, a series of composite adsorbents were synthesized with varying amidoxime/matrix ratios (35/65, 50/50, 65/35). The samples were characterized with FT-IR, XRD, SEM, EDX, XRFES spectroscopy and TGA. Under static conditions of uranium sorption, the dependence of the efficiency of radionuclide recovery from mineralized solutions of various acidities on the ratio of the initial components was established. In the pH range from 4 to 8 (inclusive), the uranium removal efficiency exceeds 95%, while the values of the distribution coefficients (Kd) exceed 10^4^ cm^3^g^−1^. It was demonstrated that an increase in the surface development of the sorbents enhances such kinetic parameters of uranium sorption as diffusion rate by 10–20 times compared to non-porous materials. The values of the maximum static capacity exceed 700 mg g^−1^. The enhanced availability of adsorption centers, achieved through the use of a porous SiO_2_ matrix, significantly improves the kinetic parameters of the adsorbents. A composite with optimal physicochemical and sorption properties (amidoxime/matrix ratio of 50/50) was examined under dynamic conditions of uranium sorption. It was found that the maximum dynamic sorption capacity of porous materials is four times greater compared to that of a non-porous adsorbent Se-init. The effective filter cycle exceeds 3200 column volumes—twice that of an adsorbent with a monolithic surface. These results indicate the promising potential of the developed materials for uranium sorption from liquid mineralized media under dynamic conditions across a wide pH range.

## 1. Introduction

The annual increase in the growth rate of the global industry directly depends on the energy capabilities needed to provide the required capacity. The use of traditional energy resources (oil, gas, coal, etc.) is severely limited both by the volume of their current production and in the long term. In addition, the burning of natural hydrocarbons is accompanied by the uncontrolled release of carbon dioxide and carbon monoxide into the atmosphere, which generally leads to an ecosystem imbalance [1].

Along with this, nuclear energy has solidified its position as a sustainable energy source over the past few decades. This recognition has been achieved through the adoption of more energy-dense fuels and decreased emissions of hydrocarbon products into the environment [2]. However, despite the proactive advancements in nuclear energy and the development of sustainable utilization techniques, several unresolved challenges remain in the industry, preventing the full realization of a closed fuel cycle. The primary component of nuclear fuel is uranium. Its utilization generates both solid and liquid radioactive waste, which contains unprocessed uranium along with its fission products. Furthermore, the extraction and processing of uranium can result in the release of radionuclides into the environment. The main migration route for uranium and its decay products is the hydrosphere. This creates risks of contamination of natural water bodies used in agriculture or even as a source of drinking water [3]. The toxic and radiotoxic dangers posed by uranium isotopes, which exist in various water-soluble forms in the biosphere, cause the need to develop new methods for extracting and concentrating uranium from natural and industrial waters with complex chemical composition, including as a valuable raw material.

To date, many different techniques for uranium removal from liquid media are known. The main methods are: extraction [4,5], electrocoagulation [6], chemical precipitation [7], membrane filtration [8], photocatalytic reduction [9], sorption [10,11,12], etc. Despite the variety of approaches available, many of them face significant challenges for uranium removal from large volumes of media. This is attributed to high energy costs [8], the generation of secondary waste [7], low selectivity, and inefficiency in complex mixtures [4,5,8], among other factors. Among all the previously described methods, the sorption technique for uranium removal stands out as the most effective and efficient due to its environmental friendliness, cost-effectiveness, high selectivity, and simplicity of the process [4,5,13,14]. Depending on the chemical composition of the solution and the conditions of sorbent use, various types of materials can act as adsorbents: inorganic [15], organometallic frameworks [16], polymers [17], carbon [18], magnetic [19], biometrics [20], etc.

A number of studies focus on the synthesis and investigation of materials based on the oxides and hydroxides of Mn [5,21], Fe [19], Ti [21,22], and Al [23] for uranium removal from various liquid media with medium and high mineralization in pH 4–8. range. The primary advantage of these materials is their developed morphology and large surface area, which offers a large number of adsorption-active sites [15]. In [24], it was suggested to utilize various types of titanium silicates, amorphous granular phosphates and mixed titanium phosphate–silicates as adsorbents for the U (VI) removal. The disadvantage of these materials is that they are limited to the removal of just the cationic forms of the radionuclide, so from aqueous media with a pH < 5.5. To expand the functionality of sorption materials and improve their sorption-selective properties, different composite materials were developed. In these materials, graphene oxides [11,25], clay minerals [26,27], or organic polymers [28,29] were used as a matrix. The deposition of metal oxides on inorganic substrates contributes to the formation of nanoscale gradient sheets of MxOy. This structure of the sorbents enhances the kinetic parameters of adsorption [26]. This approach enables an increase in sorption capacity by 1.5 times, reaching levels of 120 to 180 mg/g. However, the issue of low selectivity of these sorbents for uranium in the presence of competing ions remains significant. Analysis of the results shows that the sorption properties of manganese oxide-based sorbents towards U (VI) significantly decrease in the following order: UO_2_^2+^ > Cr^3+^ > Co^2+^ > Eu^3+^ > La^3+^ > Sr^2+^ > Zn^2+^ > Ni^2+^ [26].

From this perspective, the use of organic matrices such as polydopamine and polypyrrole, which offer a greater number of functional groups, possess reduced adhesion, and have increased reactivity [28], appears more promising. However, at the same time, the values of the zero charge point (*pH_PZC_*) of adsorbents decrease from 4.2 to 2.0. This reduction significantly limits the efficacy of the adsorbents for the highly efficient removal of the radionuclide across a wide pH range

The literature analysis indicates that composite sorbents based on organo-inorganic substrates are promising materials for uranium removal from liquid media. However, in order to preserve the advantages of individual composite components, it is necessary to develop new approaches for obtaining similar materials [30,31,32].

This work particularly focuses on sorption materials that have undergone the amidoximation stage and contain amphoteric functional groups of amidoxime containing both oxime and amino groups in the structure. This type of ligand allows the formation of stable chelate complexes with heavy metals of various types and oxidation degrees. This ligand also forms a five-membered chelate with U(VI) by transferring unshared pairs of oxygen electrons from both the oxime and amino groups to the positively-charged metal [33].

Sorption materials having amidoxime functional groups in their composition have great potential and are widely studied from an applied perspective by researchers all over the world. They have proven themselves to be highly effective adsorbents for the removal of uranium from various media (both of natural and man-made origin) with high carbonate and sulfate content. These materials are also suitable for uranium removal from seawater [34]. Promising materials are three-dimensional coordination polymers with high sorption characteristics. An example of such a structure is the Se- and S-derivatives of the 4-aminofurazan-3-carboxamidoxime with polymeric structures that were previously developed by our team [35]. The materials exhibit enhanced sorption-selective properties for uranium in liquid media across a wide pH range.

However, the fine dispersion of the adsorbents, along with their low strength and dense monolithic surface, limits their use under the harsh hydrodynamic conditions of uranium sorption. The sorption process is accompanied by blockage of the filter cartridge and peptization of the sorption material. It leads to the leaching of the sorbent into the filtrate.

In view of this, the aim of this work is to synthesize and investigate the physicochemical properties of new composite materials based on the Se derivative of 4-aminofurazane-3-carboxamidoxime and a highly porous matrix (silica gel) with enhanced mechanical strength.

## 2. Results and Discussion

### 2.1. Physicochemical Properties of the Sorbent

In this work, composite adsorbents were developed by applying a sorption-active component of the polymer type to a mechanically stable, highly porous SiO_2_ matrix.

Given that the molecular structure and microstructure of materials significantly influence uranium adsorption and characterize the adsorbent–adsorbate binding mechanism, a comprehensive study of the physicochemical properties of the synthesized composites was conducted. A non-composite adsorbent (Se-init) was used as a comparison sample [31].

**Figure 1:**
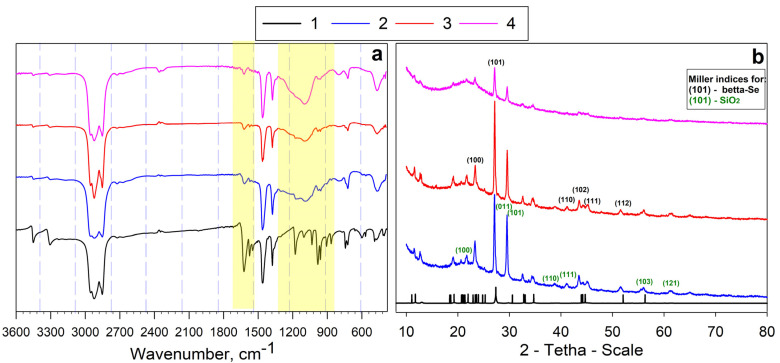
IR-spectra—(**a**) and X-ray images—(**b**) of synthesized materials: 1—Se-init, 2—Se/Si-35, 3—Se/Si-50. 4—Se/Si-65.

The IR spectrum (Figure 1a) of composite materials includes all the characteristic absorption bands of the Se-init, indicating the direct formation of the sorption-active component on the silica gel surface. This is achieved by covalently binding the amidoxime component to the active centers of silica gel, allowing for the preservation of functional radicals. An absorption band at 3454 cm^−1^ is observed in all spectra, corresponding to O–H oscillations in the oxime functional group. The absorption band at 3000 cm^−1^ correlates with H–N< oscillations, while the band at 1620 cm^−1^ is associated with C=N oscillations, and the band at 1360 cm^−1^ corresponds to C–N bond oscillations. The presence of absorption bands in the 410 cm^−1^ and 490 cm^−1^ regions corresponds to vibrations Se–Se and =Se–N< bonds, respectively. Also, an important feature is the presence of an absorption band of 1549 cm^−1^, which corresponds to fluctuations of >N–C=Se. A distinctive feature of the IR spectra of the composite materials is the broadening of peaks in the range of 900–1300 cm^–1^, which is associated with the appearance of the –Si–O–Si– bond in the range of 1015–1100 cm^−1^, corresponding to silica gel [36].

The results of XRD (Figure 1b) also indicate the presence of an amorphous SiO_2_ phase. The intensity of the corresponding reflex (18 < 2θ < 28) increases in direct proportion to the mass content of SiO_2_ in the composite. The diffraction maxima of SiO_2_ silica gel with P3(2)21 symmetry correspond to crystallographic planes with the Miller indices (100), (011), (101), (110), (012), etc. The reflexes corresponding to the crystal structure of the Se-init are predominant, which can be explained by the formation of an amidoxime film on the surface of the silica gel. The diffraction maxima observed in the experimental diffractogram correspond to crystallographic planes with Miller indices (100), (101), (110), (102), (111), and (112), as well as other characteristic diffraction maxima associated with gray selenium (β-Se) with P3(1)21 symmetry. Additionally, the diffractograms of the Se-containing materials include signals corresponding to phases of selenium that have not been previously reported in the literature.

**Figure 2:**
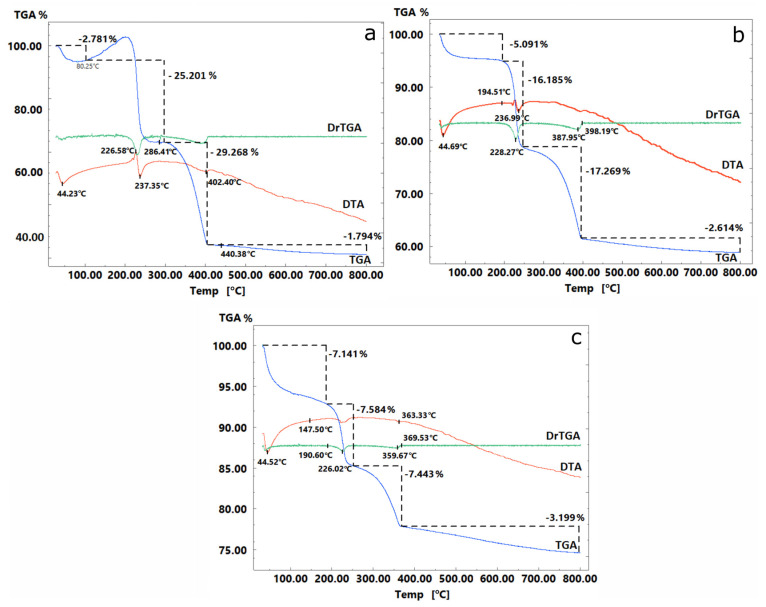
Thermograms of composite materials: (**a**)—Se/Si-35, (**b**)—Se/Si-50. (**c**)—Se/Si-65.

Thermogravimetric analysis of the materials was performed to confirm the matrix/sorption-active component ratio. The thermograms of the composite materials (Figure 2) exhibit characteristic exothermic and endothermic effects corresponding to the gradual removal of weakly bound water (up to 120 °C), which was probably absorbed by the sorbent from the air after heat treatment. Further mass loss corresponds to the polycondensation of the organic component, resulting in the removal of intracrystalline and bound water as by-products of this process. An increase in temperature to 200 °C initiates thermal oxidative degradation, leading to the decomposition of the organic component and resulting in the greatest mass loss of the sorbent.

Based on the TGA results (Figure 2), the actual ratio of the initial components in the composites was determined (Table 1). The discrepancy between the experimental and theoretical data on the adsorbent composition is probably caused by either the limited directional synthesis of the composites or incomplete combustion of carbon during TGA. However, the difference in practical values is not significant; therefore, the synthesis approach remained unchanged.

**Figure 3:**
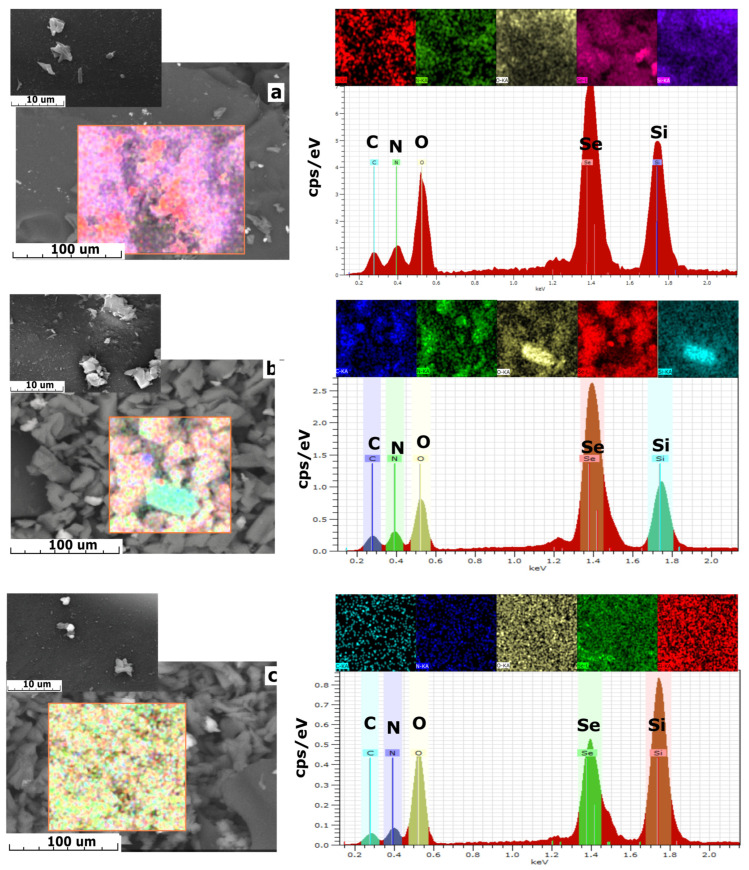
SEM images along with EDS spectra: (**a**)—Se/Si -35, (**b**)—Se/Si -50. (**c**)—Se/Si -65.

The analysis of SEM images (Figure 3) indicates that the surface of the composites exhibits a dense monolithic structure with inclusions in the form of irregular particles. According to the EDS data, the formation of initial Se-derived amidoxime occurs across the entire surface of the silicon–oxygen matrix, resulting in the formation of agglomerates in the form of large Se-derived amidoxime particles (Se-init). The number of individual particles increases with a higher mass content of amidoxime.

Despite the active filling of the matrix pore space caused by the formation of a sorption-active layer on its surface, the composite materials are characterized by a more developed surface. This is confirmed by the evaluation results of the specific pore volume and specific surface area of the sorbents (Table 2), which indicate that both values increase proportionally with the mass fraction of silica gel.

**Figure 4:**
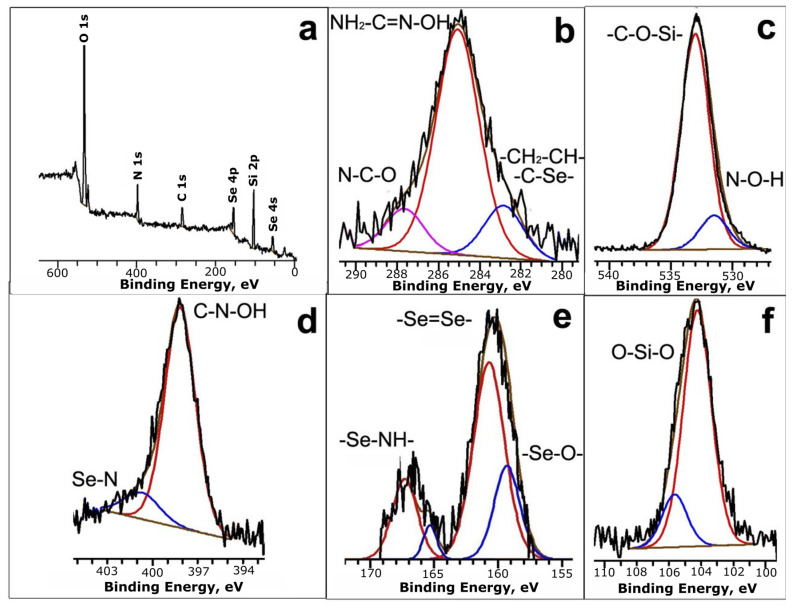
XPS spectra of non-composite Se-init: (**a**)—general view; (**b**)—C 1s region, (**c**)—O 1s region, (**d**)—N 1s region, (**e**)—Se 3p region, (**f**)—Si 2p region.

On the X-ray photoelectron (RP) spectrum, there is a maximum (285.0 eV) corresponding to a C 1s (Figure 4b), which indicates the presence of aliphatic carbon in the –CH_2_–CH– position [37]. The peak at 285.0 eV corresponds to three-substituted carbon (NH_2_-CH=N-OH). The maximum with an energy of 286.9 eV corresponds to the C=N/C–N groups. In the N 1s region (Figure 4d), there is a peak at 399.2 eV, characteristic of nitrogen in the triazine structure (HO–N–C), and a maximum at 400.4 eV corresponds to nitrogen in the thiadiazole structure, which is probably associated with the selenium (C–NH–Se). The center of the peaks in the O 1s region (Figure 4c), with energies of 533.6 eV and 531.2 eV, correspond to the OH group and oxygen bound to Si, respectively.

The position of the Se 3p peak (Figure 4e), with a binding energy of 164.0 eV, is typical for Se^4+^, which probably contributes to the formation of the polymer mesh due to the >Se=Se< bond. The spectrum also contains a peak at an energy of 169.3 eV, which corresponds to a selenium atom in a C–NH–Se surrounding. The Si 2p band in the region of high binding energies (Figure 4f) includes a contribution from the band corresponding to the SiO_2_ bond.

**Figure 5:**
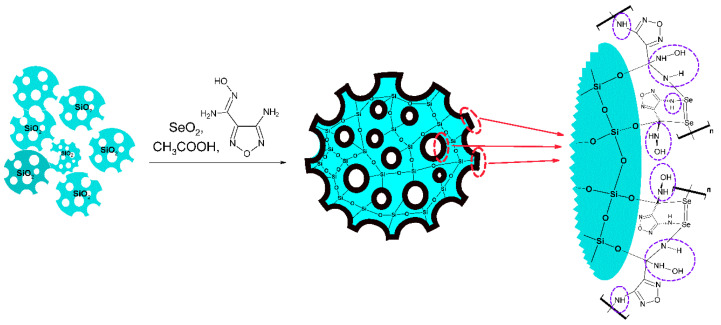
A schematic representation of the production of silica gel-based composites, along with their proposed molecular structure.

Based on the XPS analysis data, the molecular structure for the synthesized composites was proposed (Figure 5).

### 2.2. The Sorption Characteristics Under Static Conditions

**Figure 6:**
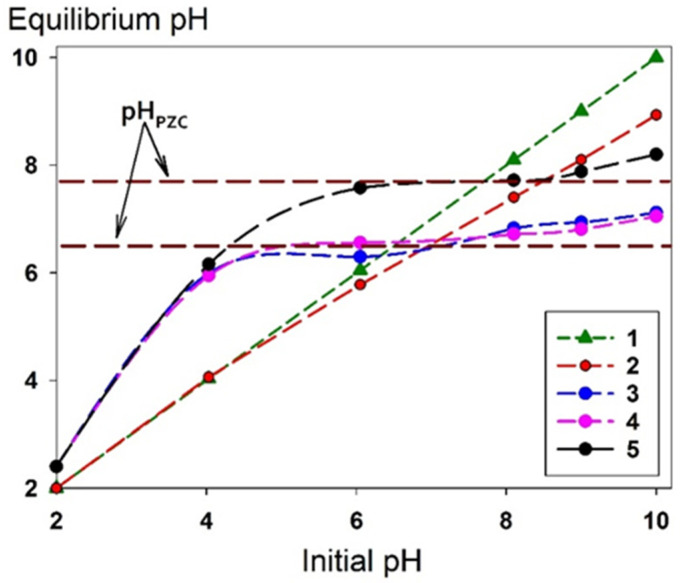
Curves of equilibrium and initial pH values for determination of *pH_pzc_*, 1—initial pH solution, 2—blank experiment (pH changing without sorbent), 3—Se/Si-35, 4—Se/Si-50, 5—Se/Si-65.

Figure 6 shows the results of the determination of the zero-charge point (*pH_PZC_*) obtained in the experiment with NaNO_3_ solution of a concentration of 0.01 mol L^−1^. The results are presented as a graphical dependence of the final equilibrium pH values of solutions after contact with a sorbent on the initial pH values of these solutions. The bending points on the curves 3, 4, and 5 (Se/Si-35, Se/Si-50, Se/Si-65, respectively) correspond to *pH_PZC_*_,_ which is the value of the solution pH, at which the sorbent surface has no charge and is in equilibrium. Composite materials are characterized by higher *pH_PZC_* values compared to Se-init. The values of the parameter were 6.0, 6.4, and 7.8 for Se/Si-35, Se/Si-50, and Se/Si-65, respectively, compared to 4.3 for Se-init. This increase is directly related to the contribution of the silicate matrix, which can function as a strongly basic ion exchanger. This capability allows for the additional binding of anionic forms of uranium in alkaline media or cationic forms in solutions with a pH of ≤7 due to the presence of ≡Si–O–. However, in acidic media, protonation becomes the predominant reaction.

**Figure 7:**
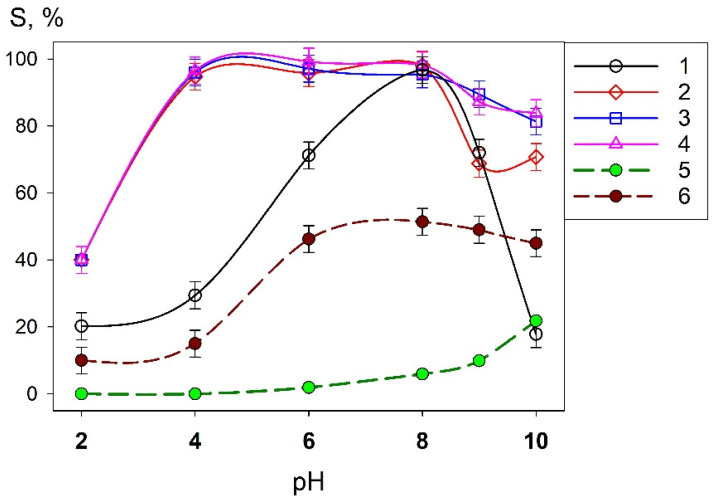
Graph of the dependence of uranium sorption efficiency on the type of model solution with pH 2–10: 1—Se-init, 2—Se/Si-35, 3—Se/Si-50. 4—Se/Si-65, 5—blank experiment, 6—silica gel matrix (V:m = 1000 mL g^−1^).

The results of the determination of *pH_pzc_* agree and allow us to establish a dependence with the results obtained when estimating the efficiency of uranium extraction from solutions with different pH (Figure 7). The solution pH was adjusted using HNO_3_ and NaOH solutions. The data presented in Figure 7 indicate that composite materials have enhanced sorption characteristics compared to Se-init. This improvement is attributed to an increase in the specific surface area of the adsorbent (Table 2), which leads to a higher number of sorption-active centers. In the pH range from 4 to 8 (inclusive), the uranium removal efficiency exceeds 95% (Figure 7), while the values of the distribution coefficients (*Kd*) exceed 10^4^ cm^3^ g^−1^. The higher adsorption efficiency of composite materials compared to Se-init, at pH values of 4, 6, 9, and 10, is probably due to the presence of a strongly basic ion exchange matrix of SiO_2_. However, as shown in curve No. 2 (Se/Si-35), the excess silica gel content limits the sorption-selective properties of materials for uranium in an alkaline medium. This limitation is due to the low sorption capacity of the material and its weak selectivity to uranium in the presence of competing ions.

**Figure 8:**
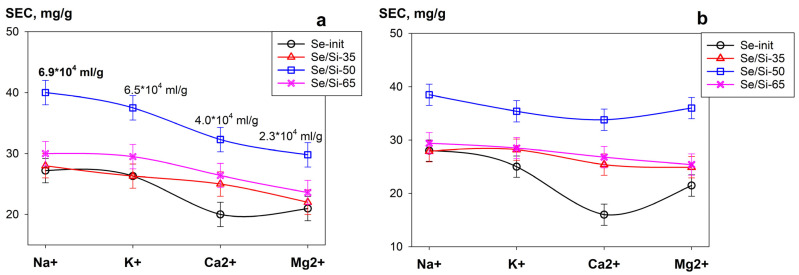
Relationship between the SEC (uranium) and the type of the competing cations in a solution (C = 0.1 mol L^−1^) with (**a**) pH 6; (**b**) pH 8 (V:m ratio = 1000 mL g^−1^).

Uranium removal is particularly crucial for solutions with high salinity and complex chemical compositions. Therefore, we evaluated the selectivity of adsorbents for the radionuclide in the presence of competing ions. Figure 8 shows the relationship between the SEC value of uranium and the types of the most common competing cations in model solutions at pH 6 and pH 8. The data indicate that an increase in electrostatic interaction during the transition from single to double-charged cations leads to a decrease in sorption capacity. Additionally, the highly competitive action of Ca^2+^ and Mg^2+^ ions may attributed to their high charge density and covalent ionic radii, which are close to that of uranium. Irrespective of the solution type, the *SEC* values decrease in the following order: Na⁺ ≤ K⁺ < Ca^2^⁺ < Mg^2^⁺. Under the conditions considered (competing ion concentration of 0.1 mol L^−1^), the K_d_ values ranged from 10⁴ to 10⁵ mL g^−1^. As the concentration of cations decreases from 0.01—0.0001 mol L^−1^, the *Kd* value increases, reaching 10^7^ mL g^−1^.

Uranium undergoes hydrolysis and exhibits an affinity for complexation, effects that are particularly pronounced in neutral and slightly alkaline environments. Consequently, the presence of various anions in solutions can significantly reduce the efficiency of uranium extraction due to the formation of uranium anionic complexes with varying degrees of oxidation. As noted in references [38,39,40], a decrease in the selectivity of uranium sorption is especially evident in the presence of carbonate and sulfate anions, which can exceed 200 mg L^−1^ and 6000 mg L^−1^, respectively, in anthropogenic or natural waters.

**Figure 9:**
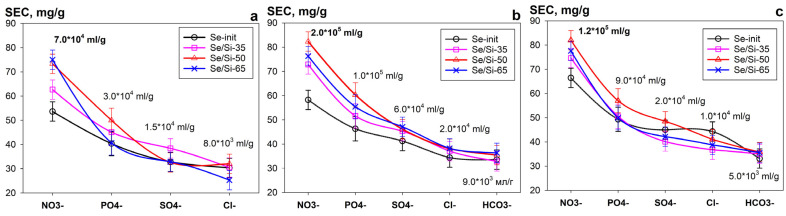
Relationship between the SEC (uranium) and the type of the competing anions in a solution (C = 0.01 mol L^−1^), (**a**)—pH 4, (**b**)—pH 6, (**c**)—pH 8 (V:m ratio = 1000 mL g^−1^).

Figure 9 shows the relationship between the *SEC* value of uranium and the type of the most common competing anions in model solutions at pH 4, pH 6 and pH 8. It was found that the anions of different natures and ionic charges negatively affected the uranium sorption value to varying degrees. The *SEC* values decrease in the following order: NO_3_^−^ < PO_4_^3−^ < Cl^−^ ≤ SO_4_^2−^ < HCO_3_^−^. This is primarily due to the formation of anionic complexes of the type [UO_2_(CO_3_)_2_(H_2_O)_2_]^2−^, [UO_2_(CO_3_)_3_]^4−^, [UO_2_(SO_4_)_3_]^4−^ [41], which exist mainly in the range at pH 6.5–11.5. When nitrate ions were replaced with bicarbonate ions (at a concentration of 0.01 mol L^−1^), the *SEC* value decreased practically twofold, and the *Kd* value decreased approximately an order of magnitude. During the transition to the acidic pH region, the sorption-selective properties of the sorbent decrease slightly, which is associated with both competing protonation reactions and the formation of complex ions of the type [UO_2_NO_3_]^+^, [UO_2_PO_4_]^–^, [UO_2_Cl]^+^, UO_2_Cl_2_^0^, etc. [42].

**Figure 10:**
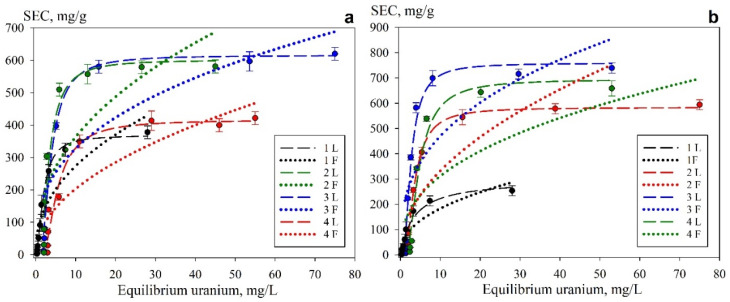
Sorption isotherms of uranium ((**a**)—pH 6, (**b**)—pH 8) and graphical approximation of the experimental values by the Langmuir equation 1L (Se-init), 2L (Se/Si-35), 3L (Se/Si-50), 4L (Se/Si-65) and by Freundlich equations 1F (Se-init), 2F (Se/Si-35), 3F (Se/Si-50), 4F (Se/Si-65).

Figure 10 shows the experimental adsorption isotherms obtained in the model solutions at pH 6 (Figure 10a) and pH 8 (Figure 10b) and the curves representing the approximation of experimental values using the Langmuir and Freundlich equations. The corresponding approximation coefficients are provided in Table 3. Visually, according to Giles [43], isotherms can be attributed to the L-type, indicating a high affinity of the adsorption centers for uranium at each pH value

The highest values of sorption capacity are observed in a model solution at pH 8 and amount to about 500–800 mg g^−1^. The loss of sorption-selective properties as the pH decreases is probably attributable to the changes in the ionic form of uranium in the solution. At pH 8, the predominant forms are UO_2_(OH)_3_^–^ along with a small amount (UO_2_)_3_(OH)_5_^+^, while at pH 6—UO_2_(OH)^+^, (UO_2_)_2_(OH)_2_^2+^, (UO_2_)_2_(OH)_2_^0^ и (UO_2_)_3_(OH)_5_^+^ [40].

The highest coefficients of determination (Table 3) were obtained using the Langmuir equation and amounted to 0.97–0.99. This indicates that the chemisorption of uranium onto the homogeneous adsorbent surface, driven by covalent binding and complexation, is the predominant mechanism for its sorption. The values of the adsorption equilibrium constant differ from each other within acceptable values in order to state that all composite materials have the same type of uranium adsorption mechanism. The highest values of the *G_max_* were obtained for Se-50 (760 mg g^−1^) and Se-65 (690 mg g^−1^), which could be related to the greater availability of sorption-active centers and a larger specific surface area (Table 2) of the adsorbents.

**Figure 11:**
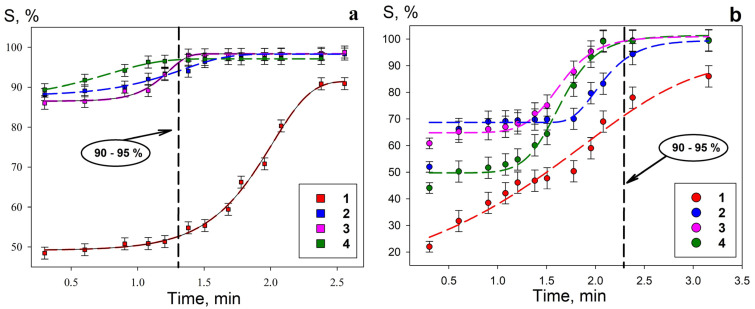
Kinetic curves of uranium adsorption from the model solution under static conditions: (**a**)—pH 6, (**b**)—pH 8; 1—Se-init, 2—Se/Si-35, 3—Se/Si-50. 4—Se/Si-65.

Figure 11 and Table 4 present the results of the kinetic parameter evaluation for uranium sorption on composite materials and Se-init. The kinetic curves demonstrate that the uranium sorption on the materials with developed surface morphology at a higher intensity correlates with an increase in the surface area of the sorption-active component. This is confirmed by a significant decrease in the half-exchange time and an increase in the diffusion coefficient values (Table 4).

The sorption kinetic parameters (Table 4) and the maximum sorption capacity (Table 3) of the composite adsorbents indicate that the optimal ratio of amidoxime to silica gel is 50/50 (Se/Si-50). The decrease in sorption-selective characteristics of Se/Si-65 is related to the low content of the sorption-active component (35% by weight). The loss of sorption characteristics in the Se/Si-35 sample is due to an excess of the amidoxime component, which blocks the pore space of the silica gel. This reduces the sorbent working surface area and, consequently, the number of sorption centers.

To estimate the rate of adsorption of uranium, Table 4 presents the values of the rate constants calculated using the pseudo-first-order and pseudo-second-order equations. Based on the determination coefficients, the kinetic curve of uranium adsorption for Se-init is better approximated by a pseudo-first-order equation, in contrast to the composite sorbents. The correspondence of the obtained results to the pseudo-second-order reaction equation for silica gel-based sorbents may indicate an increase in the availability of adsorption centers due to the developed surface morphology and the predominance of the mixed chemisorption process of U(VI) binding. Additionally, the pseudo-second-order rate constant *k_2_* (mg g^−1^ min^−1^) varies among composite sorbents. For the Se/Si-65 sorbent, the calculated _k2_ value is the lowest, which is associated with the reduced content of the sorption-active fraction (amidoxime), as reflected in the kinetic characteristic values.

### 2.3. The Sorption Characteristics Under Dynamic Conditions

**Figure 12:**
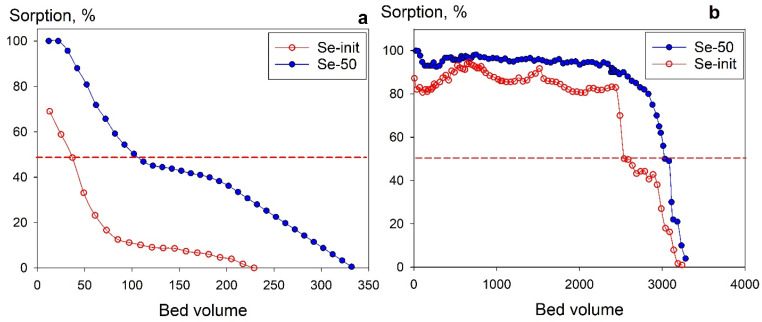
Sorption of uranium under dynamic conditions from model solutions, (**a**)—pH 6, (**b**)—pH 8.

An important feature of sorbents is their suitability for dynamic sorption conditions, which ensure the continuity of the sorption process. Figure 12 displays the uranium sorption curves for model solutions at pH 6 (Figure 12a) and pH 8 (Figure 12b) under dynamic conditions. Total dynamic exchange capacity (*TDEC*, up to 100% infiltration of uranium into the filtrate) was 0.13 mmol g^−1^ for Se-init and 0.44 mmol g^−1^ for Se/Si-50 at pH 6 and 0.54 mmol g^−1^ for Se-init and 0.84 mmol g^−1^ for Se/Si-50 at pH 8. The improved sorption characteristics of the composite sorbent, compared to Se-init, are probably due to the higher availability and increased number of adsorption centers. Percolation of the model solution (at pH 8) at a rate of 10 b.v./h through a sorption column containing 1 mL of Se-init or Se/Si-50 achieved effective cycles of filtration (50% uranium slip into the filtrate) of over 2500 and 3000 bed volumes, respectively. This performance allows us to recommend these materials for practical use. Further studies on sorption columns, focusing on determining the optimal percolation rate for model solutions of both natural and anthropogenic origin, will help establish the effective operating conditions for the adsorbents.

## 3. Conclusions

The work presents a simple approach for synthesizing new composite materials based on the Se-derivative of 4-amino-N’-hydroxy-1,2,5-oxadiazole-3-carboximidamide, implemented by forming an amidoxime component on the surface of highly porous silica gel. A series of physicochemical studies demonstrated that the formation of an amidoxime film on the surface of the matrix occurs through strong covalent bonding while preserving the organo-functional groups. Additionally, the specific pore size and surface area of the composite adsorbents increase by 15 to 100 times compared to non-porous material.

The sorption properties of the materials were characterized under static conditions of uranium sorption in the presence of various cations and anions. The materials exhibit high chemical stability and enhanced sorption-selective characteristics toward the radionuclide within the pH range of 4 to 9.

It was found that an increase in the specific surface area of sorbents using a highly porous matrix improved to an improvement in the kinetic parameters of U(VI) adsorption by an order of magnitude compared to non-porous materials. The maximum static capacity reached 760 mg g^−1^ (Se/Si-50). The increased availability of adsorption centers due to the use of a porous matrix significantly enhances the kinetic parameters of the composites, reducing the half-exchange time by 5 to 10 times compared to non-porous adsorbents. The optimal ratio of amidoxime to silica gel is 50/50 (Se/Si-50).

Under dynamic sorption conditions, a fourfold increase in the *TDEC* for uranium was observed, with the effective filter cycle reaching more than 3000 column volumes before a 50% slip occurred. Consequently, the synthesized materials can be recommended for the removal of uranium from slightly alkaline waters with high mineralization, both from technological and natural sources.

## 4. Materials and Methods

The sorption materials were synthesized using selenium dioxide, acetic acid, ethyl alcohol, and extra-pure grade methylene chloride. Commercially available silica gel, with a particle size of 35–70 mesh, was used as a matrix. For the preparation of model solutions, we used metal salts of purissimum grade without additional purification, purchased from LLC “NevaReactiv”. The radionuclide U-238 in the form of UO_2_(NO_3_)_2_ of the especially pure grade was used as a sorption element.

### 4.1. Synthesis of the Se-Derivative N’-Hydroxy-1,2,5-Oxadiazole-3-Carboximidamide

To obtain the non-composite material, 4-amino-N’-hydroxy-1,2,5-oxadiazole-3-carboximidamide was brought in contact with Se(IV) oxide in a 1:1 molar ratio. Acetic acid was added to the mixture, after which the resulting solution was boiled with intense stirring. After 80–100 min, a dark purple precipitate was formed. After the formation of the precipitate was complete, the mixture was cooled to room temperature, and the reaction product was separated from the mother liquor on a “blue ribbon” filter (pore size 2–3 microns). Unreacted components of the original mixture were removed. For this purpose, the resulting precipitate was washed Se-quantically with cold distilled water, ethyl alcohol and methylene chloride. The resulting product was dried to a constant weight for 24 h. The final material, denominated Se-init, had purple granules of a non-regular shape having a grain size of 0.05–0.2 mm [35].

### 4.2. Synthesis of Composite Sorbents

Composite materials were obtained when 4-amino-N’-hydroxy-1,2,5-oxadiazole-3-carboximidamide was brought into contact with Se(IV) oxide in a 1:1 molar ratio. Acetic acid and finely divided, highly porous silica gel were added to the mixture mixture, after which the resulting solution to the mixture and was boiled with intense stirring. After 80–100 min, a dark purple precipitate was formed. After completion of the precipitate separation, the mixture was cooled to room temperature and the precipitate was separated from the mother liquor on a “blue ribbon filter” (pore size 2–3 µm). The unreacted components of the initial mixture were removed. For this purpose, the obtained precipitate was washed successively with cold distilled water, ethyl alcohol and methylene chloride. The prepared materials comprised dark purple powders with a grain size of 0.05–0.2 mm. Three types of composite materials were synthesized; they were denominated as Se/Si-35, Se/Si-50, and Se/Si-65, with amidoxime/silica gel ratios of 65/35, 50/50, and 35/65 wt. %, respectively.

### 4.3. Evaluation of Sorption Characteristics Under Static Conditions

Sorption experiments were performed using uranium nitrate salt, where uranium is represented as U(VI). Since uranium in solutions has more than two ionic forms of existence, and the goals and objectives of the work did not include the study of the influence of ionic forms on the process of sorption of the radionuclide, all its references in work will be in the form of the designation “uranium”.

A study of sorption properties of the materials under static conditions was carried out by removal of uranium from solutions with a volume-to-mass ratio of 1000 mL g^−1^. Mixing was conducted for 24 h using an orbital shaker. The oscillation amplitude and rotation speed were 10 mm and 150 rpm, respectively. The experiment began with the sorbents being kept in a model solution without uranium for one day. After separating the solution from the sorbent, the model solution with uranium content ranging from 20 to 30 mg L^−1^ was added. After 24 h of stirring, the uranium-containing model solution was separated from the sorbent by filtration through an acetylcellulose filter with a pore size of 45 µm. The residual uranium content in the filtrate was determined using Arse-nazo III at a wavelength of 656 nm [39].

The results of the obtained uranium concentration values were used to calculate the removal efficiency (*S*, %) and uranium distribution coefficients (*Kd*, mL g^−1^) using Equations (1) and (2), respectively.

(1) $$S=\left(1-\left(\frac{C_{e}}{C_{i}}\right)\right)\times 100$$

(2) $$K_{d}=\frac{C_{i}-C_{e}}{C_{i}}\times \frac{V}{m}$$

where *C_i_*—the initial concentration of uranium in the model solution (mg L^−1^), *C_e_*—the equilibrium residual concentration of uranium in the solution after filtration (mg L^−1^), *V*—the volume of the solution (mL); *m*—the weight of the sorbent sample (g). Using the initial and equilibrium pH values of the solution, the zero charge point values (*pH_pzc_*) were calculated using the graphical method.

The influence of the presence of cations and anions in the solution on the static exchange capacity of uranium sorption was evaluated using chloride solutions of the corresponding cations (Na^+^, K^+^, Ca^2+^, Mg^2+^, Co^2+^, and Ni^2+^) or sodium salts of the corresponding cations (HCO_3_^−^, NO_3_^−^, Cl^−^, SO_4_^2−^, and PO_4_^3−^), respectively.

For a deeper evaluation of the changes in the sorption properties of adsorbents under static conditions and in the presence of competing ions, the concentration of these ions in the solution was varied from 0.001 mol L^−1^ to 0.1 mol L^−1^.

At the end of the experiment, the equilibrium uranium content in the solution was estimated to calculate the values of static exchange capacity (*SEC*, mg g^−1^) according to the following formula:

(3) $$SEC=\frac{\left(C_{i}-C_{e}\right)\times V}{m}$$

where: *Ci*—the initial concentration of uranium in the model solution (mg L^−1^), *C_e_*—the equilibrium residual concentration of uranium in the solution after filtration (mg L^−1^), *m*—the weight of the sorbent sample (g), *V*—the volume of the solution (L).

Using uranium adsorption isotherms, the nature of the adsorption process was evaluated. For this purpose, a series of samples with the masses of adsorbents ranging from 0.001 to 2 g were brought into contact with model solutions containing 0.01 mol L^−1^ NaNO_3_, and 30 mg L^−1^ uranium. The acidity of the model solutions was varied between pH 6 and 8. The experimental process involved identical processing steps as in the previous experiments. The sorption isotherms were constructed and described using the standard Langmuir (4) and Freundlich (5) equations:

(4) $$G=G_{max}\times \frac{K_{l}\times C_{e}}{1+K_{l}\times C_{e}}$$

(5) $$G=K_{f}\times C^{m}$$

where *G_max_*—the maximum sorption value (mg g^−1^), *K_l_*—the constants of the adsorption equilibrium characterizing the energy of the adsorbent–adsorbate bond (L g^−1^).

The experimental data were approximated by means of the specified equations using the SciDAVis software (ver.1.23).

The kinetic parameters of the uranium sorption process were evaluated using a model solution containing 0.1 mol L^−1^ sodium nitrate and 20–30 mg L^−1^ uranium in the form of uranyl nitrate. Two types of model solutions were used in this work, with pH 6 and pH 8. A 1 mol L^−1^ NaOH solution was used to adjust the pH of the solution. The ratio of liquid to solid phase was 200 mL g^−1^. After the adsorbent was brought into contact with the model solution, an aliquot of the solution was taken after a certain time interval to determine the residual amount of uranium in the solution. The total sorption time did not exceed 48 h.

To determine the estimate of the kinetic parameters of the ion exchange of the uranium adsorption process, the Boyd–Adamson Equation (6) [24] was utilized. This equation allows for the calculation of the effective diffusion coefficient using the values of the diffusion rate constant using the following equation:

(6) $$F=\frac{Q_{t}}{Q_{max}}=1-\frac{6}{\pi^{2}}\times e^{-Bt}$$

where: *Q_t_*—the concentration of uranium [mg L^−1^] on the sorbent at time *t*; *Q*_max_—the concentration of uranium [mg L^−1^] on the sorbent at maximum sorption, *B* is the diffusion rate constant (sec^–1^), which is described by Equation (7):

(7) $$B=\frac{\pi^{2}D_{i}}{r^{2}}$$

where: *D_i_*—the effective diffusion coefficient [cm^2^ min^−1^], *r*—the radius of the ionite grain [cm].

The dependence of *Bt* as a mathematical function on *F* is described by Equation (8) and is given in the form of tabular values by Richenberg in [44].

(8) $$Bt=6.28318-3.2899F-6.28318({1-1.0470)}^{\frac{1}{2}},\;\;at\;F\leq 0.85$$

where, *B_t_*—Fourier homochrony criteria.

The value of *B* was determined as the tangent of the angle of inclination of the straight line obtained by approximating the initial values of the kinetic curve (for *F* < 0.5) in the coordinates *Bt = f(t).* With the value of B known, Di was calculated using Equation (7).

Additionally, the obtained kinetic curves in S_(t)_—t coordinates were processed by the pseudo-first-order (9) and pseudo-second-order (10) kinetic equations.

(9) $$A_{t}=A_{e}\times (1-{exp}^{-k_{1}\times t})$$

(10) $$A_{t}=\frac{k_{2}\times {A_{e}}^{2}\times t}{1+k_{2}\times A_{e}\times t}$$

where *A_t_*—the amount of adsorbed uranium at time *t* [mmol g^−1^], *A_e_*—the equilibrium uranium adsorption [mmol g^−1^], *t*—time [min], and *k_1_* and *k_2_* are the pseudo-first-order and pseudo-second-order constants, respectively.

### 4.4. Evaluation of Sorption Characteristics Under Dynamic Conditions

The study of the dynamic characteristics of sorption was conducted under continuous flow conditions using a model solution with a pH of 6 or 8 and 0.1 mol L^−1^ NaNO_3_. The solution was passed through a fixed sorbent layer with a volume of 1 mL and a grain size of 0.05–0.2 mm at a rate of 10 b.v. h^−1^. Prior to this, the materials were soaked in a model solution that did not contain uranium.

Filtrates after the column were divided into fractions, and the uranium content was measured for each fraction. The sorption capacity (SP) of the materials was calculated using the Equation (11):

(11) $$S_{U}={(C}_{i}-C_{e})\times \frac{V}{m}$$

where: *C_i_*—initial uranium concentration [mol L^−1^], *C_e_*—the equilibrium uranium concentration [mol L^−1^], *V*—the model solution volume [L], m—weight of the sorbent suspension [g].

TDEC was calculated by integrating the curves that represent the dependence of adsorption on the volume of solution passed, as shown in Equation (12):

(12) $$TDEC=\int_{0}^{n}f\left(\frac{\left(C_{0}-C_{i}\right)V_{i}}{m}\right)dV_{t}$$

where: *C*_0_—the initial concentration of uranium in the model solution, [mmol mL^−1^]; *C*_i_—the concentration of uranium in *i* eluate fraction, [mmol mL^−1^]; *V*_i_—the volume of *i* eluate fraction, [mL]; *m*—sorbent weight, [g]; *V*_t_—the total solution volume, [mL].

### 4.5. Equipment

To establish the peculiarities of the molecular structure formation of the samples, a combination of physicochemical methods was employed. The determination of the types of chemical bonds of the polymer network was performed by infrared spectroscopy using a Spectrum 1000 spectrometer manufactured by Perkin Elmer (Waltham, MA, USA) in KBr pellets.

To determine the phase composition of the obtained materials and to study the nature of the interaction between the matrix and the sorption-active component, X-ray diffraction patterns were recorded using a Colibri X-ray diffractometer manufactured by JSC IC Burevestnik (Nizhniy Novgorod, Russia), with CuKα-radiation in the angle range of 2° < 2θ < 90° in the point-by-point scanning mode.

Investigations of the morphology of the surface of the obtained composites were performed using high-resolution scanning electron microscopy with a HITACHI TM 3000 device (Tokyo, Japan) at accelerating voltages of 5–15 kV and a beam current of I ≈ 100 pA. The device was equipped with an accessory for energy dispersion analysis (EDA) by Bruker (Ettlingen, Germany).

The specific surface area and pore size of the material were determined by a low-temperature nitrogen adsorption technique using an Autosorb IQ device by Quantachrome Instruments (Boynton Beach, FL, USA). The calculation was performed according to the BET method (the theory of Brunauer–Emmett–Teller).

The control of the elemental composition of model samples for studies of sorption-selective properties of materials was performed using the atomic absorption flame spectroscopy using a Thermo Solar AA M6 device manufactured by Thermo Electron Corporation (Waltham, MA, USA). The content of uranyl ions in the solution was determined by the spectrophotometric method using a Shimadzu UV-1800 spectrophotometer (Kyoto, Japan) in the wavelength range from 600 to 800 nm. The calibration curve for uranium content had a radionuclide concentration range from 0.1 mg L^−1^ to 30 mg L^−1^.

## Figures and Tables

**Table 1:** Theoretical and experimental characteristics of the synthesized materials.

	Theoretical Data		Experimental Data	
	Mass Content of Se-Init, %	Mass Content of SiO_2_, %	Mass Content of Se-Init, %	Mass Content of SiO_2_, %
Se-init	100	0	100	0
Se/Si-35	65	35	60	40
Se/Si-50	50	50	45	55
Se/Si-65	35	65	27	73

**Table 2:** Results of determination of the specific pore volume and specific surface area of the sorbents using the nitrogen adsorption method.

Parameter	Se-init	Se/Si-35	Se/Si-50	Se/Si-65
Silica gel content, wt. %	0	35	50	65
Pore volume, cm^3^ g^−1^	0.05	0.45	0.51	0.65
Specific surface area, mL g^−1^	2	210	243	298
Specific pore size, nm	1.21	12.1	12.1	12.1

**Table 3:** Theoretical values of the constants of the Langmuir and Freundlich equations calculated after approximating the experimental uranium sorption data.

	Equation	Parameter	Se-init	Se/Si-35	Se/Si-50	Se/Si-65
pH-6	Langmuir	G_max_, mgg^−1^	370 ± 20	420 ± 20	620 ± 20	600 ± 30
		K_l_	0.24 ± 0.05	0.14 ± 0.07	0.14 ± 0.05	0.14 ± 0.07
		R^2^	0.988	0.968	0.989	0.949
	Freundlich	K	100 ± 20	108 ± 39	141 ± 57	66 ± 27
		n	0.440 ± 0.080	0.487 ± 0.116	0.368 ± 0.111	0.490 ± 0.118
		R	0.820	0.785	0.753	0.801
pH-8	Langmuir	G_max_, mg g^−1^	460 ± 10	580 ± 20	760 ± 30	690 ± 20
		K_l_	0.30 ± 0.07	0.19 ± 0.05	0.18 ± 0.07	0.21 ± 0.09
		R^2^	0.98	0.99	0.97	0.98
	Freundlich	K	67 ± 14	124 ± 46	206 ± 73	107 ± 55
		n	0.436 ± 0.080	0.400 ± 0.104	0.359 ± 0.112	0.490 ± 0.153
		R	0.831	0.757	0.752	0.736

**Table 4:** Kinetic and sorption-selective parameters of uranium adsorption from model solutions at pH 6 and pH 8.

№	Parameters	pH 6				pH 8			
		Se-init	Se/Si-35	Se/Si-50	Se/Si-65	Se-init	Se/Si-35	Se/Si-50	Se/Si-65
1	t_max_ × 10^−2^ min	2.4	0.48	0.24	0.48	14.4	14.4	2.4	14.4
2	K_d_ × 10^−5^, cm^3^ g^−1^	0.03	6.1	6.7	6.8	0.067	0.32	0.32	0.32
3	Di × 10^6^, cm^2^ min^−1^	2.9	31.4	58.0	23.3	5.3	4.7	12.7	10.4
4	T_1/2_, min	58.0	5.4	2.9	28.2	32.2	36.1	13.3	16.3
5	R^2^	0.992	0.989	0.995	0.992	0.972	0.950	0.993	0.996
	Kinetic parameters of the model for the pseudo-first-order and pseudo-second-order equations								
6	k_1_, min^−1^, (PFO)	0.02	0.07 **	0.08 **	0.06 **	0.11	0.68 **	0.75 **	0.55 **
7	k_2_, mg g^−1^ min^−1^, (PSO)	0.9**	6.6	8.1	4.2	5.0**	12.7	13.9	10.4

PFO—pseudo-first-order kinetic equations; PSO—pseudo-second-order kinetic equations. **—The values of the rate constants for the pseudo-first-order and pseudo-second-order models, along with a correlation coefficient of less than 0.90.

## Data Availability

All data and materials are available on request from the corresponding author. The data are not publicly available due to ongoing researches using a part of the data.
